# Synthesis of a novel brominated vinylic fatty acid with antileishmanial activity that effectively inhibits the *Leishmania* topoisomerase IB enzyme mediated by halogen bond formation

**DOI:** 10.1515/pac-2018-1113

**Published:** 2019-03-06

**Authors:** Néstor M. Carballeira, Denisse Alequín, Leilani M. Lotti Diaz, Victorio Jauregui Matos, Leonardo L. G. Ferreira, Adriano D. Andricopulo, Mikhail Y. Golovko, Rosa M. Reguera, Yolanda Pérez-Pertejo, Rafael Balaña-Fouce

**Affiliations:** University of Puerto Rico, Río Piedras Campus, 17 Ave Universidad STE 1701, San Juan, PR 00925-2537, USA, Tel.: (787)-764-0000 ext, 88561; Department of Chemistry, University of Puerto Rico, Río Piedras Campus, San Juan, PR, USA; Laboratory of Medicinal and Computational Chemistry, Center for Research and Innovation in Biodiversity and Drug Discovery, Physics Institute of Sao Carlos, University of Sao Paulo, Sao Carlos, SP 13563-120, Brazil; Department of Biomedical Sciences, University of North Dakota School of Medicine and Health Sciences, 1301 N Columbia Road, Grand Forks, ND 58202-9037, USA; Department of Biomedical Sciences, University of León, Campus de Vegazana, León 24071, Spain

**Keywords:** brominated fatty acids, halogen bond, *Leishmania infantum*, leishmaniasis, NTD2018, synthesis, topoisomerase IB

## Abstract

Many marine derived fatty acids, mainly from sponges, possess vinylic halogenated moieties (bromine or iodine) but their assessment as antileishmanial candidates remains elusive. In this work, we undertook the first total synthesis of a novel series of 2-allyl-3-halo-2-nonadecenoic acids, which preferentially inhibit the *Leishmania* DNA topoisomerase IB enzyme (*L*TopIB) over the human topoisomerase IB enzyme (hTopIB). The synthesis of 2-allyl-3-bromo-2*E*-nonadecenoic acid (**1a**) and 2-allyl-3-chloro-2*E*-nonadecenoic acid (**2a**) was achieved through a palladium catalyzed haloallylation of 2-nonadecynoic acid (2-NDA) using either allyl bromide or allyl chloride in the presence of PdCl_2_(PhCN)_2_ in 57–83 % overall yields. Among the new halogenated synthetic compounds, **1a** was the most inhibitory of *L*TopIB with an EC_50_ = 7 μM, while the shorter chain analogs 2-allyl-3-bromo-2*E*-dodecenoic acid (**1b**) and 2-allyl-3-chloro-2*E*-dodecenoic acid (**2b**), synthesized from 2-dodecynoic acid, were not inhibitory of *L*TopIB (EC_50_ > 100 μM) resulting in the overall order of inhibition **1a** > 2-NDA > **2a** > > **1b** ≅ **2b**. The acids **1a** and **2a** inhibit *L*TopIB by a Gimatecan-independent mechanism. The enhanced *L*TopIB inhibition of **1a** was computationally rationalized in terms of a halogen bond between the bromine in **1a** and a DNA phosphate (binding energy = − 4.85 kcal/mol). Acid **1a** also displayed preferential cytotoxicity towards *Leishmania infantum* amastigotes (EC_50_ = 2.5 μM) over *L. infantum* promastigotes (EC_50_ > 25 μM).

## Introduction

The British Medical Journal has classified Leishmaniasis as one of the “main neglected tropical diseases” [1]. *Visceral leishmaniasis* is a tropical zoonotic disease caused by species of intracellular parasites of the Leishmania genus. *Leishmania donovani* and *Leishmania infantum* in the Old World and *Leishmania chagasi* in the New World invade spleen and liver macrophages, causing anemia and fever and ultimately death if not properly treated [2]. There are between 50 000 and 90 000 cases each year, mostly in countries like India, Bangladesh, Brazil, Ethiopia, and the Sudan [3]. Among the present therapeutic venues to combat the disease we count on pentavalent antimonials such as meglumine antimoniate, the antifungal amphotericin B or the antibacterial paromomycin, and oral chemotherapy drugs such as miltefosine [4]. Combinations of these drugs is an urgent response to the lack of new chemical identities against this disease [3]. The problem continues to be the nephrotoxicity of the drugs and the emerging resistance by the parasite [4].

Important to mention here is that the life cycle of the parasite contains two main forms, i.e. the promastigote and amastigote stages [5]. Sand flies typically inject promastigotes into the victim during a blood meal and neutrophils phagocytize the promastigotes by rapidly moving to the bite site. Infected neutrophils then release the parasites, and the phagolysosomes of vertebrate macrophages internalized them. Inside macrophages, the promastigotes transform into amastigotes, which can then multiply in the cells of various tissues, the so-called diagnostic stage. A blood meal by a sand fly from an infested individual again results in the ingestion of the macrophages into the fly where the amastigotes transform again in the midgut into promastigotes to start the cycle again [3].

One validated therapeutic target of *Leishmania* parasites, that our research group has been particularly interested, is the DNA topoisomerase IB (*L*TopIB) enzyme [6], [7]. To unwind DNA, type I-B topoisomerases form a phosphotyrosine linkage with the 3′ end of the DNA cut strand, while type I-A topoisomerases form the phosphotyrosine linkage with the 5′ end. However, *L*TopIB is phylogenetically unique, it has an anomalous dimeric structure, and it lends itself for therapeutic intervention because of its difference with the human DNA topoisomerase IB (hTopIB) enzyme [6].

There is ample precedence in the literature that fatty acids inhibit both hTopIB and *L*TopIB. Work by Majumder and Desideri revealed that fatty acids inhibit hTopIB by a mechanism different from the one displayed by ternary complex stabilizers such as camptothecin (CPT) [8]. For example, the researchers concluded that conjugated eicosapentaenoic acid (cEPA) does not bind to the DNA-topoisomerase binary complex, but rather interacts directly with hTopIB. The acid binds in the whereabouts of the active site and essentially blocks the DNA cleavage by the catalytic tyrosine Y723 and prevents it from executing the nucleophilic attack on the DNA phosphate. The authors also indicated that cEPA inhibits the human enzyme only upon pre-incubation of the compound with the enzyme [8].

Our research group has been particularly active in studying *L*TopIB inhibition by fatty acids, but emphasizing novel fatty acids of natural origin. For example, the natural occurring 6-icosynoic acid, isolated from *Sommera sabiceoides*, inhibits *L*TopIB with an EC_50_=49 μM [9], while the synthetic fatty acid 16-phenyl-6-hexadecynoic acid inhibits *L*TopIB with an EC_50_=14 μM [10]. α-Methoxylated fatty acids, such as the (±)-2-methoxy-6-heptadecynoic acid, also display good inhibition of the enzyme (EC_50_=16.6 μM) [11]. In some cases, *L*TopIB inhibition is a good predictor of antileishmanial activity, but there is no correlation between these two inhibitory activities. For example, while the 6-icosynoic acid displays toxicity towards *L. donovani* promastigotes at an EC_50_=3.6 μM [9], the acid (2*R*,5*Z*,9*Z*)-2-methoxy-25-methyl-5,9-hexacosadienoic acid, which inhibits *L*TopIB with an EC_50_=11.5 μM, only displays toxicity towards *L. infantum* amastigotes at an IC_50_=0.17 mg/mL [12]. Noteworthy to mention is that most of these acids do also inhibit hTopIB, but for the shorter-chain acids there is a strong preference for *L*TopIB over hTopIB. For example, while the 16-phenyl-6-hexadecynoic acid inhibits *L*TopIB with an EC_50_=14 μM, it inhibits hTopIB at an EC_50_=25 μM [10]. This tendency is reversed for the longer-chain fatty acids as exemplified by the (2*R*,5*Z*,9*Z*)-2-methoxy-25-methyl-5,9-hexacosadienoic acid, which inhibits hTopIB at an EC_50_=4.6 μM [12]. In addition, in most of the examples that we have examined so far, we have also observed that fatty acids tend to be more toxic towards *L. infantum* amastigotes than towards *L. infantum* promastigotes.

Fatty acids not examined so far for antileishmanial activity are the halogenated fatty acids. Many of these lipids are of marine origin [13], [14], [15]. For example, brominated and iodinated fatty acids were identified in several species of sponges and anemones. Common are fatty acids with the 6-bromo-5*E*,9*Z*-diene functionality. For example, the acid (5*E*,9*Z*)-6-bromo-5,9-eicosadienoic acid was isolated from the anemone *Condylactis gigantea* [13], while acids such as the (5*E*,9*Z*)-6-bromo-23-methyl-5,9-tetracosadienoic acid were identified in the sponge *Agelas* sp. [14]. A similar 6-iodo-5*E*,9*Z*-diene functionality was identified in novel acetylenic acids from the sponges *Suberites mammilaris* and *Suberites japonicas* [15].

While the antileishmanial activities of the former halo acids have not been elucidated, we hypothesized that acids with a brominated vinylic functionality could be interesting as TopIB inhibitors for the potential of forming halogen bonds with either the enzyme or the DNA. Halogen bonds are widely used in medicinal chemistry and there are several studies that utilize halogen substitution to improve drug performance [16], [17]. For example, a recent study demonstrated that 2α-bromo-dihydrobelulonic acid is a better inhibitor of the human topoisomerase IIα than betulinic acid while displaying an improved cytotoxicity against HeLa cells at a significantly lower IC_50_=7.5 μM as compared to that of betulinic acid (IC_50_=30 μM) [16].

With this end goal in mind, we synthesized the novel 2-allyl-3-bromo-2*E*-nonadecenoic acid (**1a**) and 2-allyl-3-chloro-2*E*-nonadecenoic acid (**2a**) as well as the corresponding C12 analogs **1b** and **2b** and tested their potential as *L*TopIB and hTopIB inhibitors together with their antileishmanial activity towards *L. infantum* amastigotes. We report that the presence of the bromine substituent in **1a** greatly enhanced its topoisomerase inhibitory activity as well as its antiparasitical activity towards *L. infantum* amastigotes.

## Materials and methods

The analysis of all the compounds was performed by ^1^H NMR (300 and 500 MHz) and ^13^C NMR (75 and 125 MHz) using either a Bruker DPX-300 or a Bruker Avance DRX-500 spectrometer. ROESY spectra were measured on a Bruker Avance DRX-400 spectrometer. The samples were dissolved in 99.8% chloroform-d (CDCl_3_), the solvent signals at 7.26 (^1^H) and 77.0 (^13^C) ppm were used as internal standards for hydrogen and carbon, respectively. Mass spectral data was acquired on a single quadrupole GC-MS (Agilent 7820/Agilent 5977E) equipped with a 30 m×0.32 mm (film 0.25 μm) capillary column (DB-5MS) of phenyl arylene polymer which is virtually equivalent to a (5%-phenyl)-methylpolysiloxane. The IR spectra were measured neat on a Bruker Tensor 27 FT-IR spectrometer or on a Thermo Nicolet 1S5.

Fatty acid UPLC-MS analysis was performed using an ACQUITY UPLC pump coupled to Synapt G2-SQ-TOF mass spectrometer (Waters, Milford, MA, USA). One μg of fatty acid in 1 μg of 2-propanol was loaded on an ACUITY UPLC HSS T3 column (1.8 μm, 100 Å pore diameter, 2.1×150 mm, Waters) and resolved using a liner gradient of solvent B that was increased from 35% to 99% over 9 min. Solvent B was acetonitrile/ 2-propanol (10:90) containing 10 μM ammonium acetate and 0.02% acetic acid. Solvent A consisted of acetonitrile/water (40:60) with 10 μM ammonium acetate and 0.025% acetic acid. The mass spectrometer was operated in a negative electrospray ionization mode. MS^E^ sensitivity mode was used to collect data with the low energy of 2V, and the collision energy in the T-wave element ramped from 10 to 25V. Leucine enkephalin (400 pg/μL) was infused at a rate of 10 μL/min for mass correction. MassLynx V4.1 software (Waters) was used for instrument control, acquisition, and sample analysis.

### Synthesis of 2-allyl-3-bromo-2E-nonadecenoic acid (1a)

To a mixture of 0.457 g (1.55 mmol) of 2-nonadecynoic acid and bis(benzonitrile)palladium(II) chloride (0.078 g, 0.5 mmol, 5 mol%) 13.4 mL (155 mmol) of allyl bromide were added dropwise under a N_2_ atmosphere at 0°C. The solution was left stirring at room temperature (rt) for 12 h. The remaining allylbromide was removed under reduced pressure. The crude product was purified using silica gel column chromatography eluting with hexane/ether (95:5) affording **1a** (0.325 g, 0.78 mmol) as a white solid (mp 56–59°C) in an 83% yield. UV (hexane) λ_max_ (log ε) 243 nm (4.13); IR (neat) ν_max_: 3500–2500 (O–H), 2954, 2916, 2846, 1685 (C=O), 1638 (C=C), 1612 (C=C), 1463, 1434, 1407, 1283, 1227, 918, 722, 640 (C-Br), 608 cm^−1^; ^1^H NMR (CDCl_3_, 500 MHz) δ (ppm) 5.83 (1H, ddt, J_trans_=17 Hz, J_cis_=10 Hz, J_vic_=6.1 Hz, H-2′), 5.14 (1H, ddt, J_trans_=17.2 Hz, J_gem_=1.7 Hz, J_allyl_=1.6 Hz, H-3′trans), 5.07 (1H, ddt, J_cis_=10.1 Hz, J_gem_=1.7 Hz, J_allyl_=1.3 Hz, H-3′cis), 3.33 (2H, brd, H-1′), 2.98 (2H, brt, H-4), 1.65 (2H, m, H-5), 1.26 (26H, s, H-6 to H-18), 0.87 (3H, t, *J=*7.0 Hz, -CH_3_); ^13^C NMR (CDCl_3_, 125 MHz) δ (ppm) 171.22 (s, C-1), 147.24 (s, C-3), 133.28 (t, C-3′), 129.30 (s, C-2), 116.37 (d, C-2′), 40.42 (t), 38.83 (t), 34.67 (t), 31.93 (t), 31.59 (t), 29.71 (t), 29.67 (t), 29.65 (t), 29.50 (t), 29.37 (t), 29.33 (t), 28.92 (t), 28.76 (t), 22.69 (t), 22.65 (t), 14.11 (q). UPLC-HRMS (negative ion mode) *m/z* [M-1]^+^ Calcd for C_22_H_38_O_2_Br 413.2055; Found 413.2047.

#### Methyl 2-allyl-3-bromo-2E-nonadecenoate

GC-MS (70 eV) *m/z* (rel intensity) 430 (M^+^+2, 1), 428 (M^+^, 2), 349 (M^+^-Br, 100), 317 (M^+^-Br-MeOH, 6), 289 (8), 220 (6), 218 (7), 153 (5), 135 (9), 121 (13), 107 (14), 91 (12), 81 (14), 79 (22), 67 (12), 55 (20).

### Synthesis of 2-allyl-3-chloro-2E-nonadecenoic acid (2a)

To a mixture of 0.30 g (1.02 mmol) of 2-nonadecynoic acid and bis(benzonitrile)palladium(II) chloride (0.19 g, 0.5 mmol, 5% mol) 8.29 mL (102 mmol) of allyl chloride were added dropwise under a N_2_ atmosphere at 0°C. The solution was left stirring at this temperature for 12 h. The crude mixture was then filtered through Celite to remove the catalyst and the solvent rotoevaporated in vacuo. The crude product was then purified using silica gel column chromatography eluting with hexane/ether (95:5) affording **2a** (0.217 g, 0.58 mmol) as a white solid (mp 42–46°C) for a 57% yield. UV (hexane) λ_max_ (log ε) 235 nm (4.22); IR (neat) ν_max_: 3500–2500 (O–H), 2956, 2917, 2849, 1686 (C=O), 1633 (C=C), 1610 (C=C), 1464, 1435, 1406, 1284, 1229, 1129, 1053, 990, 918, 722, 670 (C–Cl) cm^−1^; ^1^H NMR (CDCl_3_ 300 MHz) δ (ppm) 5.85 (1H, ddt, J_trans_=17 Hz, J_cis_=10 Hz, J_vic_=6.1 Hz, H-2′), 5.10 (2H, m, J_trans_=17 Hz, J_cis_=10 Hz, J_gem_=1.7 Hz, J_allyl_=1.6 Hz, H-3′), 3.31 (2H, brd, H-1′), 2.88 (2H, brt, H-4), 1.65 (2H, m, H-5), 1.30 (26H, s, H-6 to H-18), 0.87 (3H, t, *J*=6.7 Hz, -CH_3_); ^13^C NMR (CDCl_3_, 125 MHz) δ (ppm) 171.68 (s, C-1), 153.30 (s, C-3), 133.57 (t, C-3′), 126.58 (s, C-2), 116.10 (d, C-2′), 38.03 (t), 35.51 (t), 34.37 (t), 31.91 (t), 29.69 (t), 29.65 (t), 29.49 (t), 29.35 (t), 29.32 (t), 29.04 (t), 28.89 (t), 28.69 (t), 28.20 (t), 27.89 (t), 22.68 (t), 14.10 (q). UPLC-HRMS (negative ion mode) *m/z* [M-1]^+^ Calcd for C_22_H_38_O_2_Cl 369.2560; Found 369.2569.

#### Methyl 2-allyl-3-chloro-2E-nonadecenoate

GC-MS (70 eV) *m/z* (rel intensity) 370 (M^+^, 2), 336 (23), 335 (M^+^-Cl, 100), 317 (M^+^-Cl -MeOH, 16), 289 (10), 193 (10), 175 (27), 173 (50), 160 (48), 139 (16), 137 (29), 135 (16), 124 (36), 107 (21), 91 (36), 81 (39), 79 (64), 67 (34), 55 (63).

### Synthesis of 2-allyl-3-bromo-2E-dodecenoic acid (1b)

To a mixture of 0.75 g (3.82 mmol) of 2-dodecynoic acid and bis(benzonitrile)palladium(II) chloride (0.073 g, 0.19 mmol, 5 mol%), 33.0 mL (380 mmol) of allyl bromide were added dropwise under a N_2_ atmosphere at 0°C. The reaction was warmed up to rt and left to react for 12 h. The crude was filtered through Celite to remove the catalyst and the solvent was removed under vacuum. The product was purified over silica eluting with hexane and ethyl acetate (7:3) to afford **1b** (0.57 g, 1.80 mmol) as a yellow oil in a 42% yield. IR (neat) ν_max_: 3700–2700 (O–H), 3082, 2929, 2858, 1695 (C=O), 1651 (C=C), 1598 (C=C), 1460, 1407, 1280, 1224, 1130, 1082, 992, 915, 724, 641 (C–Br) cm^-1^; ^1^H-NMR (CDCl_3_, 300 MHz) δ (ppm) 5.86 (1H, ddt, J_trans_=17 Hz, J_cis_=10 Hz, J_vic_=6.2 Hz, H-2′), 5.11 (2H, m, J_trans_=17 Hz, J_cis_=10 Hz, J_gem_=1.7 Hz, J_allyl_=1.6 Hz, H-3′), 3.32 (2H, brd, H-1′), 2.89 (2H, brt, H-4), 1.67 (2H, m, H-5), 1.28 (12H, s, H-6 to H-12), 0.91 (3H, t, J=6.7 Hz, -CH_3_); ^13^C-NMR (CDCl_3_, 75 MHz) δ (ppm) 171.04 (s, C-1), 147.06 (d, C-3), 133.58 (t, C-3′), 129.35 (s, C-2), 116.37 (d, C-2′), 40.40 (t), 38.84 (t), 31.86 (t), 29.43 (t), 29.31 (t), 29.27 (t), 28.92 (t), 28.74 (t), 28.20 (t), 14.07 (q).

### Synthesis of 2-allyl-3-chloro-2E-dodecenoic acid (2b)

To a mixture of 0.75 g (3.82 mmol) of 2-dodecynoic acid and bis(benzonitrile)palladium(II) chloride (0.073 g, 5 mol%) 33.0 mL (380 mmol) of allyl chloride were added dropwise under a N_2_ atmosphere at 0°C. The solution was stirred at this temperature and warmed up to rt for 12 h. The crude material was purified directly over silica gel eluting with hexane and ethyl acetate (9:1) to afford **2b** (0.76 g, 0.78 mmol) as a yellow/orange oil in a 73% yield. IR (Neat) ν_max_: 3700–2700 (O–H), 3082, 2954, 2923, 2853, 1683 (C=O), 1640 (C=C), 1602 (C=C), 1456, 1437, 1405, 1281, 1225, 1123, 1039, 990, 915, 722, 669 (C–Cl) cm^−1^. ^1^H-NMR (CDCl_3_ 300 MHz) δ (ppm) 5.85 (1H, ddt, J_trans_=17 Hz, J_cis_=10 Hz, J_vic_=6.1 Hz, H-2′), 5.10 (2H, m, J_trans_=17 Hz, J_cis_=10 Hz, J_gem_=1.7 Hz, J_allyl_=1.6 Hz, H-3′), 3.32 (2H, brd, H-1′), 2.90 (2H, brt, H-4), 1.67 (2H, m, H-5), 1.30 (12H, s, H-6 to H-12), 0.91 (3H, t, *J*=6.6 Hz, H-12).^13^C-NMR (CDCl_3_, 75 MHz) δ (ppm) 171.59 (s, C-1), 153.15 (d, C-3), 133.59 (t, C-3′), 126.65 (s, C-2), 116.10 (d, C-2′), 38.02 (t), 35.53 (t), 31.86 (t), 29.43 (t), 29.31 (t), 29.26 (t), 28.88 (t), 28.20 (t), 25.28 (t), 14.06 (q).

### *Leishmania infantum* studies

#### Leishmania infantum iRFP strain

The biological procedures with living animals were undertaken according to Spanish Act (RD 53/2013) and EU legislation (2010/63/EU). All protocols were approved by the Animal Care Committee of the University of León (Spain) Project license number PI12/00104. The generation of the infrared-fluorescent strains was described previously by the authors in the literature [18], [19]. *Leishmania infantum* BCN-150 was modified with the pLEXSY-hyg2 vector (Jena Bioscience, Germany), fused to the IRFP protein and the 3′UTR region of histone Hsp70 from *Leishmania*, which increases the expression of the proteins of *L. infantum*. The iRFP strain was subcultured at a density of 10^6^ cells/mL in a previously described medium [10]. These cultures were placed in 96-well microplates (Nunc, Fisher Sci), aliquoted in 200 μL volumes per well, and challenged at the different concentrations of the fatty acids, which were incubated at 26°C. An ODYSSEY^®^ (Li-Cor) imaging equipment was used to record the infrared signal emitted at 708 nm by the cultures after 52 h. The SigmaPlot 10.0 program was used to process the data obtained by a non-linear curve fitting to obtain the IC_50_ values.

Primary splenic cell cultures were acquired from 8 to 10 week BALB/c mice inoculated i.p. with 10^8^ metacyclic *L. infantum* iRFP promastigotes. After 5 weeks of post-infection, mice were euthanized and spleens were aseptically removed. To obtain a suspension of individual cells, spleens were cut into small pieces, and incubated in collagenase D (2 mg/mL) as previously described [10]. This suspension was gently passed through a 100 μm cell strainer aided by freshly prepared PBS. Splenocytes were washed twice by centrifugation at 500 *g* for 7 min at 4°C and resuspended in RPMI (Gibco) supplemented with 10% (v/v) FCS, 1 mM sodium pyruvate (Gibco), 1×RPMI vitamins and antibiotics. After cell counting in a Neubauer chamber, splenocytes were placed in 96-well microplates at a density of 5×10^5^ cells per well and incubated at 37°C in 5% CO_2_. Macrophages were incubated for 24 h to allow their perfect adhesion to the plaque [20]. After this time, cells were challenged with different concentrations of each fatty acid over a period of 72 h. Amastigote viability within the macrophages was assessed by the infrared signal emitted at 708 nm by the cultures using an ODYSSEY® (Li-Cor) imaging equipment. The data obtained were processed by a non-linear curve fitting with the Sigma Plot 10.0 program. These experiments were done in duplicates.

### Expression and purification of recombinant Leishmania and human TopIB proteins

Cloning, expression, and purification of recombinant *L*TopIB and hTopIB proteins were performed using a *Saccharomyces cerevisiae* EKY3 TopIB-deficient strain platform and low-pressure chromatographic methods as previously reported [21]. Yeast were collected by centrifugation at 10 000 rpm and resuspended in 20 mL of TEEG buffer (50 mM Tris-HCl pH 7.4, 1 mM EDTA, 1 mM EGTA, 10% glycerol) supplemented with 0.2 M KCl and a protease inhibitors cocktail (Roche Farma SA, Spain). The protein extracts obtained after the lysis of yeast cells were loaded onto a column packed with heparin sepharose resin (GEHealthcare) and eluted with TEEG supplemented with 0.8 M KCl for hTopIB and 0.6 M for *L*TopIB.

### TopIB relaxation of supercoiled activity

The human or leishmania recombinant TopIB relaxation of supercoiled DNA was assayed by measuring the intensity of the different topoisomers with distinct electrophoretic mobility in an agarose gel as previously described [10]. Briefly, the reaction took place in a final volume of 20 μL, 2 μL of TOP buffer (100 mM Tris-HCl buffer pH 7.5, 50 mM MgCl_2_, 1 mM EDTA, 150 μg/mL bovine serum albumin) 180 mM KCl and 0.5 μg of purified hTopIB or *L*TopIB. The fatty acids were initially preincubated at 4°C for 15 min with the assay mixture lacking DNA. Reactions were started by adding the substrate; 0.5 μg of supercoiled circular pBluescript-SK DNA. Incubations were carried out in Eppendorf tubes for 30 min at 37°C. Reactions were stopped by the addition of 1% SDS (w/v) and digested with 1 mg/mL proteinase K at 37°C to remove contaminating protein linked to DNA. Topoisomers were resolved by agarose gel (1% w/v) electrophoresis in TBE (Tris-borate buffer, pH 8.0) at 2 V/cm for 16 h, and stained with an ethidium bromide solution (0.5 μg/100 mL) to obtain digital images using a G- Box (Syngene UK) gel-doc.

### Competition assays with Gimatecan (GMT)

To determine if the fatty acids were covalently linked to DNA, an agarose cleavage assay was undertaken as previously described [10]. To a reaction mixture (20 μL) containing 2 μL of TOP buffer, 0.5 μg pBluescript-SK DNA, and 75–100 U of *L*TopIB or hTopIB, 1 μL of the different concentrations of the acids was added. For the competition assays a fixed concentration of 100 μM of GMT – a CPT analogous that cleaves DNA – was used [22]. The mixtures were incubated at 25°C for 10 min. After stopping the reactions with 1% (w/v) SDS, they were digested with 2 μg proteinase K for 1 h at 37°C. The samples were then extracted with a 1:1 (v/v) mixture of phenol-chloroform. Finally, the extracted samples were resolved on 1% (w/v) agarose gels in TBE (1×) supplemented with ethidium bromide at a final concentration of 40 pg/μL. Electrophoresis was performed as previously described and the bands were quantified with the ImageJ (National Institutes of Health software) gel analysis software. The amount of nicked DNA was calculated as the percentage of signal obtained for the indentation band divided by the total signal of the lane. The results were analyzed with the Sigma Plot TM 11.3 statistical software (Systat Software Inc., UK).

### Cytotoxicity on uninfected murine splenocytes and determination of the Selectivity Index (SI)

BALB/c mice were euthanized to obtain the spleen under sterile conditions. Splenocytes were extracted as previously described, seeded in 96-well plates at a cell density of 2×10^5^ cells/well and exposed to different concentrations of the tested compounds for 72 h at 37°C in 5% CO_2_. The viability of the obtained splenocytes was established using the Blue Alamar dye (Invitrogen) micromethod whereby the fluorescence emitted at 595 nm was recorded using a multimodal Synergy HT (Bio-Tek) microplate reader. The Selectivity Index (SI) was determined as the ratio between the IC_50_ values obtained for non-infected mouse splenocytes and the EC_50_ values for amastigotes measured in infected splenocytes.

### Molecular modeling

The 3D structures of the acids were built using the default geometric and atomic parameters of SYBYL-X 2.1. The 3D conformation of each compound was minimized using Tripos force field and Powell conjugate gradient algorithm with a minimum energy change of 0.005 kcal/molÅ [28], [29]. Gasteiger-Hückel charges were generated by applying a distance-dependent dielectric function and the dielectric constant of water [25]. The X-ray structure of *L*TopIB was retrieved from the Protein Data Bank (PDB 2B9S, 2.27 Å) and the DNA cleaved strand was joined to produce a noncovalent complex. The structure was relaxed using GROMACS 5.1.4 and the AMBER99SB force field [26], [27]. Water molecules were excluded prior to molecular docking and the enzyme binding site was defined as a 14 Å-radius sphere centered on Lys352. GOLD 5.3 was used in the molecular docking runs and the binding solutions were evaluated with GoldScore [28]. Each ligand was docked 20 times and only the best docking solution for each ligand (highest scoring conformation) were considered in the analyses. The protein-ligand complexes were visually examined with Pymol 1.31 [29]. Jaguar 9.3 implemented in Maestro (release 2016-3) and the OPLS force field was used to predict the energetics of the halogen bond. The structure was minimized with the OPLS3 force field and DFT calculations were run using the hybrid functional M06-2X [30], [31].

## Results and discussion

### Synthesis of the 2-allyl-3-halo-2-nonadecenoic acids 1a and 2a

**Scheme 1:**
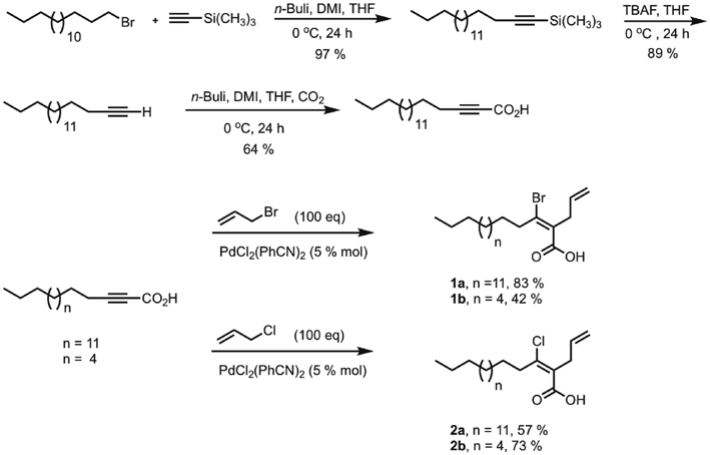
Total synthesis of the 2-NDA (above) and of the fatty acids **1a**, **1b**, **2a** and **2b** (below).

The synthesis of **1a** and **2a** started with 2-nonadecynoic acid (2-NDA), which is not commercially available and it had to be synthesized. For the synthesis of 2-NDA (Scheme 1) we used a modified version of a previously reported procedure [32]. The synthesis started with commercially available ethynyltrimethylsilane, which was coupled with 1-bromohexadecane using n-butyllithium (n-BuLi), tetrahydrofuran (THF) and 1,3-dimethyl-2-imidazolidinone (DMI) as co-solvents at 0°C. The resulting silylated alkyne was deprotected with tetrabutylammonium fluoride (TBAF) in THF at 0°C to obtain 1-octadecyne that was subsequently reacted with n-BuLi in THF followed by quenching with CO_2_ and final protonation with NH_4_Cl to afford the known 2-NDA in a 55% overall yield.

For the bromoallylation and chloroallylation of 2-NDA we followed a modified version of Topolovcan’s procedure (Scheme 1) [33]. First, 2-NDA was bromoallylated using 100 equivalents of allyl bromide and 5 mol% of bis(benzonitrile)palladium(II) chloride. The reaction was carried out between 0°C and rt for 12 h. The 2-allyl-3-bromo-2*E*-nonadecenoic acid (**1a**) was obtained in an 83% yield. A 2D HMBC (heteronuclear multiple bond correlation) experiment was necessary to unequivocally determine the regioselectivity of the halopalladation. The 2D HMBC spectrum showed a strong correlation between the C-1 carbon in **1a** and the allylic H-1′ hydrogens of the side chain. This correlation corresponded to a ^3^J_(C−H)_ consistent with the allyl group being α to the carbonyl group. The latter result discards the possibility that the allyl group could be located at the β position because in this case there would be no 2D HMBC correlation between the C-1 carbonyl carbon and the allylic H-1′ protons since that distance would have corresponded to a ^4^J_(C−H)_, which is not observed in a 2D HMBC experiment. In addition, to assign the stereochemistry of the C2–C3 alkene as *Z* or *E* a 2D ROESY (rotating frame nuclear overhauser effect spectroscopy) NMR experiment was performed. It was important to determine if there was a correlation between the allylic H-1′ protons in the side chain and the allylic H-4 protons in the main aliphatic chain, which would have implicated a *Z* stereochemistry for the double bond. Our calculations (Pymol 1.31) indicated that the H-1′ protons and the H-4 protons in **1a** are separated by a distance of 2.5 Å, well within the range of a typical NOESY or ROESY interaction, which call for distances between 1.85 Å and 3.2 Å [34]. The 2D ROESY spectrum showed no such correlation and, therefore, the alkene stereochemistry in **1a** was assigned as *E*.

For the chloroallylation of 2-NDA the same synthetic procedure as described above was used, but this time with allyl chloride as the halogen source (Scheme 1). The 2-allyl-3-chloro-2*E*-nonadecenoic acid (**2a**) was synthesized in a 57% overall yield. The synthesis of the shorter chain analogs 2-allyl-3-bromo-2*E*-dodecenoic acid (**1b**) and 2-allyl-3-chloro-2*E*-dodecenoic acid (**2b**) also followed the same synthetic strategy outlined above (Scheme 1). In the latter case **1b** was obtained in a 42% overall yield, while **2b** was obtained in a 73% overall yield. All the synthesized compounds displayed similar spectral characteristics.

### Top I inhibition studies

As a first step, we determined the Top I inhibitory activities of 2-NDA as well as that of the novel halogenated fatty acids **1a**, **1b**, **2a**, and **2b**. As the baseline for this study we determined the *L*TopIB and hTopIB inhibitory properties of 2-NDA, which displayed a half maximal effective concentration (EC_50_) of 10.3±1.1 μM towards *L*TopIB and 20.5±0.9 μM towards hTopIB, respectively (Table 1). It was of no surprise the better affinity of 2-NDA towards *L*TopIB over hTopIB, something that we have observed before for other 2-alkynoic fatty acids [35]. The inhibitory potential of **1a** significantly increased towards both enzymes as compared to 2-NDA. Presumably, the bromine substituent in **1a** is engaging in additional halogen bonding interactions with oxygens or nitrogens at the active site of the TopIBs or with oxygens of the phosphates of the DNA. In contrast to **1a**, the chlorinated analog **2a** was not as inhibitory towards the enzymes when compared to either 2-NDA or **1a**. Acid **2a** inhibited hTopIB with an EC_50_=49.7±0.0 μM, but it did better towards *L*TopIB with an EC_50_ of 25.7±2.5 μM (Table 1). While **2a** was more inhibitory towards *L*TopIB than towards hTopIB, it was less inhibitory overall than 2-NDA. Taken together, our data indicates that the addition of a bromine substituent increased the inhibitory capability of the acids towards both topoisomerases, but chlorine was not as effective as the following inhibitory order **1a**>2-NDA>**2a** was observed.

**Table 1:** Inhibition of hTopIB and *L*TopIB by 2-NDA and the halogenated fatty acids **1a** and **2a** (*N*=2).

Fatty acid	*L*TopIB EC_50_ (μM)	hTopIB EC_50_ (μM)
**2-NDA**	10.3±1.1	20.5±0.9
**1a**	7.4±0.2	12.7±0.0
**2a**	25.7±2.5	49.7±0.0
**1b**	>100	>100
**2b**	>100	>100

Reducing the carbon chain length of the acids from 19 carbons to 12 carbons was detrimental for the Top I inhibitory activities displayed by these halogenated fatty acids (Table 1). For example, **1b**, the C12 analog of **1a**, was not effective towards both Top I enzymes (EC_50′s_>100 μM). Changing the bromine substituent for a chlorine substituent, like in **2b**, also proved of no value in increasing the inhibitory potential of this short-chain analog towards both TopIBs. These results denoted that chain length is also a critical factor for the novel halogenated fatty acids to be effective topo I inhibitors. This could be rationalized in terms of less van der Waals interactions of the shorter-chain acids as compared to the longer-chain acids within the active site of the enzyme together with a different binding mode as the molecular modeling results will show.

**Fig. 1:**
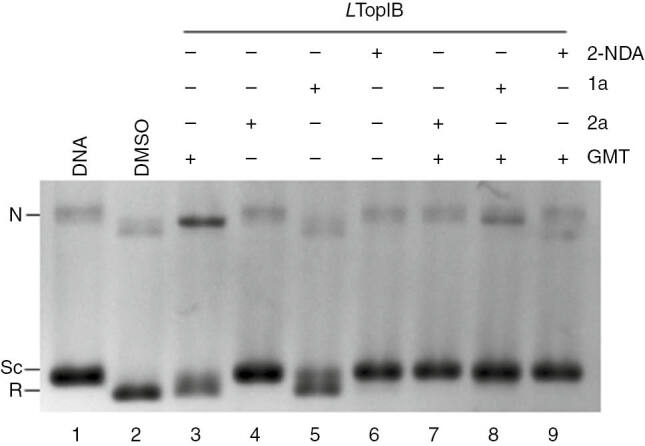
Fatty acids inhibit *L*TopIB by a GMT-independent mechanism. Purified *L*TopIB (lanes 2–9) was assayed in the presence of DMSO (lane 2), 100 μM GMT (lane 3), 100 μM **2a** (lane 4), and 100 μM **1a** (lane 5). To assess the potential competition with GMT, 100 μM of either **1a** or **2a** were added simultaneously to 100 μM GMT (lanes 7 and 8, respectively). Supercoiled, relaxed and nicked DNA were separated resolved in 1% agarose gel containing ethidium bromide to a final concentration of 40 μg/mL (N, nicked DNA; Sc, supercoiled DNA; R, relaxed DNA).

To assess if **1a** and **2a** only inhibit *L*TopIB or stabilize the cleavage complexes with DNA, as CPT and their analogs do, an agarose assay with ethidium bromide (EtBr) was undertaken (Fig. 1). In this assay, the cleavage complexes stabilized by the acids, Top IB, and DNA, were digested with proteinase K and the nicked DNA was resolved in the gel in a band appearing at the top of the lane (Fig. 1). For **1a** or **2a** we were not able to observe a nicked DNA, which was observed for the Top IB poison GMT. In a subsequent experiment either **1a** or **2a** were incubated together with 100 μM GMT to check whether these compounds compete with GMT for the same topo I active site. The agarose gel of Fig. 1 shows that the band corresponding to nicked DNA was effectively reduced by both acids, as well as the band corresponding to relaxed DNA. These results indicated that **1a** and **2a** share the same binding site as GMT, but did not stabilize the cleavage complexes between topo I and DNA.

### Molecular modeling studies

**Fig. 2:**
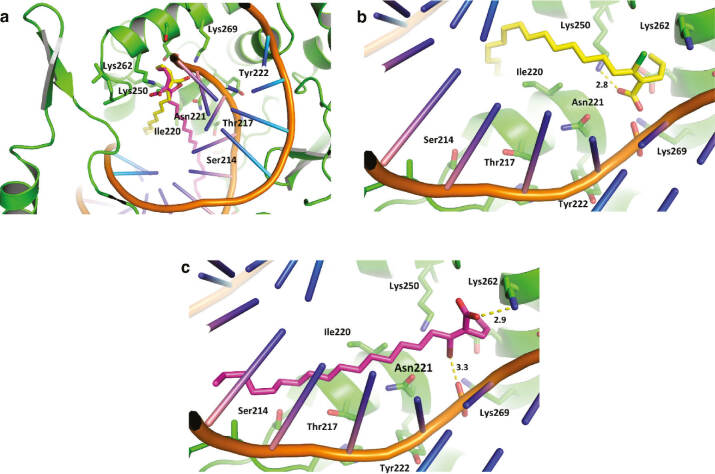
(a) Binding of **1a** and **2a** in an interface located between the active site of *L*TopIB and a major DNA major groove. (b) Highest scoring conformation of **2a** (yellow), showing an ionic interaction between the ligand carboxylate group and the side chain of Lys250. (c) Highest scoring conformation of **1a** (violet), showing an ionic interaction between the ligand carboxylate group and the side chain of Lys262, and a halogen bond involving the bromine atom and a DNA phosphate group.

To further understand the mechanism of *L*TopIB inhibition by **1a** and **2a** molecular modeling was performed. Molecular docking runs on *L*TopIB showed that the ligands bind to an interface between the DNA and the enzyme at the active site, next to the DNA cleavage point and the catalytic Tyr222. This region is surrounded by Lys269, Lys262, Lys250, Ile220, Thr217, Asn221, and Ser214 (Fig. 2a). Although not interacting directly with Tyr222, the proximity of the inhibitors to the DNA cleavage site potentially produces perturbations in nearby residues that are important for catalysis, hindering, therefore, the DNA cleavage. The polar head of the inhibitors positioned between the phosphodiester-deoxyribose backbone of the DNA and a predominantly polar enzyme subsite surrounded by Lys269, Lys262, and Lys250. The alkyl chain of the inhibitors was observed to project into a cavity formed along several α-helices and a major DNA groove. The importance of the alkyl chain of the ligands was reflected in the docking scorings, which were highly dominated by van der Waals interactions. This finding was confirmed by also docking **1b**, which failed in reproducing the binding mode of the other halogenated compounds and displayed no activity against the enzyme. The carboxylate group of **2a** (IC_50_=25.7 μM) formed an ionic interaction with Lys250, while no interaction involving the Cl atom was detected (Fig. 2b). On the other hand, **1a** (IC_50_=7.4 μM) formed a halogen bond (binding energy=−4.85 kcal/mol) with a DNA phosphate group and an ionic interaction involving the ligand carboxylate and the Lys262 side chain (Fig. 2c). The formation of the Br-phosphate halogen bond supports the higher potency of the brominated analog **1a** over the chlorinated compound **2a**.

### Antiprotozoal studies

The best candidates identified in our *L*TopIB enzymatic studies, namely 2-NDA, **1a** and **2a**, were also selected for further antiprotozoal evaluation towards *L. infantum* promastigotes and amastigotes. Among the studied acids, **1a** was the most toxic towards *L. infantum* amastigotes with an IC_50_=2.5±2.0 μM (Table 2). On the other hand, 2-NDA presented the second-best toxicity towards *L. infantum* amastigotes with an IC_50_=11.7±2.9 μM. The overall cytotoxicity observed was **1a**>2-NDA>**2a**, which correlated quite well with the observed TopIB inhibition studies. Interesting to note is that these fatty acids displayed a higher toxicity towards amastigotes than towards promastigotes. These findings once again confirm that fatty acids can distinguish between the different stages of the leishmanial parasite (amastigotes vs. promastigotes) and display differential toxicities towards each stage. This correlates with previous studies on *Leishmania mexicana* where amastigotes displayed a 10-fold higher increased uptake rate of non-esterified fatty acids compared to the promastigotes of the same species [36], [37]. The results clearly demonstrated that the bromine substituent in **1a** increased the toxicity towards *L. infantum* amastigotes with a good Selectivity Index (SI>20.3). These results also showed that chlorine substitution was not critical for the observed activities.

**Table 2:** Toxicity of 2-NDA, **1a** and **2a** against *L. infantum* promastigotes and amastigotes.

FA	Promastigotes IC_50_ (μM)	Amastigotes IC_50_ (μM)	Cytotoxicity^a^ EC_50_ (μM)	SI promastigotes	SI amastigotes
**2-NDA**	50.6±5.2	11.7±2.9	>250	>4.9	>21.4
**1a**	>25	2.5±2.0	>50	2	>20.3
**2a**	>250	18.5±5.4	>250	1	>13.5

SI, Selectivity Index. ^a^Uninfected Splenocytes from primary cultures of spleen cells. IC_50_ values are the mean of two measurements±standard deviation.

Even though there was a good correlation between the TopIB inhibition studies and the inhibitory activities displayed by **1a** towards *L. infantum* amastigotes, we should caution that our studies do not necessarily imply that TopIB inhibition is the only mechanism whereby **1a** might be killing the parasites. Other mechanisms of toxicity could very well be operative such as alterations to the membrane lipid content, disruption of either the ether lipid metabolism or the glycosylphosphatidylinositol (GPI) anchor biosynthesis which could also affect signal transduction [38], [39]. Ether lipids are important plasma membrane anchor moieties for Leishmania since these anchor essential glycolipids and glycoproteins such as gp63 [39]. Further studies on the mechanism of action of these important lipids could shed more light into their definite mode of action.

## Conclusions

This work outlines the importance of halogenated vinylic fatty acids as a new class of antiprotozoal agents against *L. infantum* amastigotes with relatively good selectivity indexes. The brominated acid **1a** was the best antileishmanial candidate in the series (IC_50_=2.5 μM), which effectively inhibits *L*TopIB (EC_50_=7.4 μM) but with the potential to disturb the ether lipid metabolism of the parasite as well as the plasma membrane anchor mechanisms. The mechanism of *L*TopIB inhibition displayed by **1a** and **2b** is different from that of ternary complex stabilizers such as Gimatecan, while the enhanced inhibitory potential of **1a** was computationally rationalized through a halogen bond between the bromine and the phosphates of the DNA. All the studied fatty acids preferentially inhibit *L*TopIB over hTopIB and carbon chain length is a critical factor for both *L*TopIB inhibition and antiprotozoal activity. This is the first time that halogenated fatty acids have been screened as antileishmanial agents thus opening the door to other analogs, with a similar unusual mode of action, that takes advantage of a halogen bond formation.

## References

[1] Yamey G., Hotez P (2007). BMJ.

[2] Murray H. W., Berman J. D., Davies C. R., Saravia N. G (2005). The Lancet.

[3] Burza S., Croft S. L., Boelaert M (2018). Lancet.

[4] Ponte-Sucre A., Gamarro F., Dujardin J. C., Barrett M. P., López-Vélez R., García-Hernández R., Pountain A. W., Mwenechanya R., Papadopoulou B (2017). PLoS Negl. Trop. Dis..

[5] Gossage S. M., Rogers M. E., Bates P. A (2003). Int. J. Parasitol..

[6] Balaña-Fouce R., Álvarez-Velilla R., Fernández-Prada C., García-Estrada C., Reguera R. M (2014). Int. J. Parasitol. Drugs Drug Resist..

[7] D’Annessa I., Castelli S., Desideri A (2015). Mini Rev. Med. Chem..

[8] Castelli S., Campagna A., Vasallo O., Tesauro C., Fiorani P., Tagliatesta P., Oteri F., Falconi M., Majumder H. K., Desideri A (2009). Arch. Biochem. Biophys..

[9] Carballeira N. M., Cartagena M. M., Prada C. F., Rubio C. F., Balaña-Fouce R (2009). Lipids.

[10] Carballeira N. M., Morales-Guzmán C., Álvarez-Benedicto E., Torres-Martínez Z., Delgado Y., Griebenow K. H., Tinoco A. D., Reguera R. M., Pérez-Pertejo Y., Carbajo-Andrés R., Balaña-Fouce R (2018). Curr. Top. Med. Chem..

[11] Carballeira N. M., Cartagena M., Li F., Chen Z., Prada C. F., Calvo-Álvarez E., Reguera R. M., Balaña-Fouce R (2012). Pure Appl. Chem..

[12] Carballeira N. M., Montano N., Amador L. A., Rodríguez A. D., Golovko M. Y., Golovko S. A., Reguera R. M., Álvarez-Velilla R., Balaña-Fouce R (2016). Lipids.

[13] Carballeira N. M., Reyes M (1995). J. Nat. Prod..

[14] Carballeira N. M., Emiliano A (1993). Lipids.

[15] Hwang B. Su, Lee K., Yang C., Jeong E. Ju, Rho J.-R. (2013). J. Nat. Prod..

[16] Ghosh S., Mukhopadhyay S., Sarkar M., Mandal A., Das V., Kumar A., Giri B (2017). Chem. Biol. Interact..

[17] Wilcken R., Zimmerman M. O., Lange A., Joerger A. C., Boeckler F. M (2013). J. Med. Chem..

[18] Calvo-Álvarez E., Stamatakis K., Punzón C., Álvarez-Velilla R., Tejería A., Escudero-Martínez J. M., Pérez-Pertejo Y., Fresno M., Balaña-Fouce R., Reguera R. M (2015). PLoS Negl. Trop. Dis..

[19] Chander S., Ashok P., Reguera R. M., Perez-Pertejo M. Y., Carbajo-Andres R., Balaña-Fouce R., Gowri Chandra Sekhar K. V., Sankaranarayanan M. (2018). Exp. Parasitol..

[20] Calvo-Álvarez E., Guerrero N. A., Álvarez-Velilla R., Prada C. F., Requena J. M., Punzón C., Llamas M. Á., Arévalo F. J., Rivas L., Fresno M., Pérez-Pertejo Y., Balaña-Fouce R., Reguera R. M (2012). PLoS Negl. Trop. Dis..

[21] Villa H., Otero-Marcos A. R., Reguera R. M., Balaña-Fouce R., García-Estrada C., Pérez-Pertejo Y., Tekwani B. L., Myler P. J., Stuart K. D., Bjornsti M. A., Ordóñez D (2003). J. Biol. Chem..

[22] Prada C. F., Álvarez-Velilla R., Balaña-Fouce R., Prieto C., Calvo-Álvarez E., Escudero-Martínez J. M., Requena J. M., Ordóñez C., Desideri A., Pérez-Pertejo Y., Reguera R. M (2013). Biochem. Pharmacol..

[23] Powell M. J. D (1977). Math. Program..

[24] Clark M., Cramer R. D., Van Opdenbosch N. (1989). J. Comput. Chem..

[25] Tsai K. C., Chen Y. C., Hsiao N. W., Wang C. L., Lin C. L., Lee Y. C., Li M., Wang B (2010). Eur. J. Med. Chem..

[26] Van Der Spoel D., Lindahl  E., Hess B., Groenhof G., Mark A. E., Berendsen H. J (2005). J. Comput. Chem..

[27] Lindorff-Larsen K., Piana S., Palmo K., Maragakis P., Klepeis J. L., Dror R. O., Shaw D. E (2010). Proteins.

[28] Verdonk M. L., Cole J. C., Hartshorn M. J., Murray C. W., Taylor R. D (2003). Proteins,.

[29] Lill M. A., Danielson M. L (2011). J. Comput. Aid. Mol. Des..

[30] Jorgensen W. L., Maxwell D. S., Tirado-Rives J (1996). J. Am. Chem. Soc..

[31] Bochevarov A. D., Harder E., Hughes T. F., Greenwood J. R., Braden D. A., Philipp D. M., Rinaldo D., Halls M. D., Zhang J., Friesner R. A (2013). Int. J. Quantum Chem..

[32] Morbidoni H. R., Vilchèze C., Kremer L., Bittman R., Sacchettini J. C., Jacobs W. R. (2006). Chem. Biol..

[33] Topolovcan N., Panov I., Kotora M (2016). Org. Lett..

[34] Andersen N. H., Eaton H. L., Lai X (1989). Mag. Reson. Chem..

[35] Carballeira N. M., Cartagena M., Sanabria D., Tasdemir D., Prada C. F., Reguera R. M., Balaña-Fouce R (2012). Bioorg. Med. Chem. Lett..

[36] Hart D. T., Coombs G. H (1982). Exp. Parasitol..

[37] Coombs G. H., Hart D. T., Capaldo J (1983). J. Antimicrob. Chemother..

[38] Vincent I. M., Weidt S., Rivas L., Burgess K., Smith T. K., Ouellette M (2014). Int. J. Parasitol. Drugs Drug Resist..

[39] Lux H., Hart D. T., Parker P. J., Klenner T, Nigam  S., Kunkel G., Prescott S. M. (1996). Platelet-Activating Factor and Related Lipid Mediators 2. Advances in Experimental Medicine and Biology.

